# Hydroxychloroquine, azithromycin and methylprednisolone and in hospital survival in severe COVID-19 pneumonia

**DOI:** 10.3389/fphar.2022.935370

**Published:** 2022-09-27

**Authors:** Ronaldo C. Go, Themba Nyirenda

**Affiliations:** ^1^ Hackensack Meridian School of Medicine, Nutley, NJ, United States; ^2^ Hackensack University Medical Center, Hackensack, NJ, United States; ^3^ Department of Critical Care, Robert Wood Johnson Barnabas Health, Hamilton, NJ, United States

**Keywords:** COVID-19, methylprednisolone, d-dimer, hydroxychloroquine, azithromycin

## Abstract

**Introduction:** Severe COVID-19 pneumonia has two phases that are not mutually exclusive. Repurposed drugs target only one phase and the association of combination therapy to survival is unknown.

**Objective:** To determine the association of hydroxychloroquine, azithromycin, and methylprednisolone versus methylprednisolone only to in hospital survival.

**Methods:** This is a secondary analysis of a retrospective cohort of patients admitted for severe covid-19 in 13 hospitals in New Jersey, United States from March–June 2020. Propensity score match with 11 variables was constructed between those who received no methylprednisolone and methylprednisolone. Multivariate Cox regression was used for risk of in hospital mortality.

**Measurements and main results:** There were 759 patients, 380 in no methylprednisolone and 379 with methylprednisolone. Multivariate Cox regression shows that methylprednisolone, hydroxychloroquine, and azithromycin had prolonged survival compared to methylprednisolone alone [HR 0.45 (95% CI 0.22,0.91 *p* < 0.03)]. In patients who received hydroxychloroquine and azithromycin, those who also received high dose methylprednisolone were associated with worse survival compared to those who received low dose methylprednisolone (HR = 1.642; 95% CI 1.053 to 2.562; *p* = 0.0287). Nursing home residents [HR 2.77 (95% CI 1.67, 4.59 *p* < 0.0001)], coronary artery disease [HR 2.93 (95% CI 1.31, 3.15 *p* = 0.001), and invasive mechanical ventilation [HR 3.02 (95% CI 1.71,5.34 *p* = 0.0001)] were independently associated with worse survival.

**Conclusion:** Combination therapy was associated with improved survival compared to monotherapy. However, nursing home residents, coronary artery disease, and mechanical ventilation were independently associated with mortality. Larger randomized controlled studies are needed to confirm conclusions.

## Introduction

After the first Coronavirus disease 2019 (COVID-19) pandemic surge in June 2021, there were 170,812,850 cases, 3,557,586 deaths, and mortality rate of 2% (World Health Organization, 2021a). In March 2022, there has been 452,201,564 confirmed cases, 6,029,852 deaths, and a mortality rate of 1.33% (World Health Organization, 2021b). The most common cause of mortality is acute respiratory distress syndrome (ARDS) (Badu et al., 2021). Drugs were repurposed to meet the time of need (Go, 2021). Azithromycin, a macrolide antibiotic, and hydroxychloroquine, an antimalarial drug, have been repurposed due to reported effects *in vitro*, readily available, and are inexpensive. Hydroxychloroquine has *in vitro* activity against SARS-CoV-2 through impairment of terminal glycosylation of angiotensin converting enzyme receptor 2 (ACE2) and increase pH of endosomes that virus use for entry. (Chen et al., 2004; Vincentet al., 2005; Liu et al., 2020; Wang et al., 2020; Yao et al., 2020; Zhou et al., 2020). Azithromycin also has *in vitro* activity against SARS-CoV-2 and hypothesized to cause cytokine downregulation, maintain epithelial integrity, and prevent pulmonary fibrosis (Echeverría-Esnal et al., 2021). The efficacy *in vitro* did not necessarily translate *in vivo*, and azithromycin and hydroxychloroquine were not associated with improved survival in several studies (Recovery Collaborative Group et al., 2020; Rosenberg et al., 2020; Fiolet et al., 2021; Réa-Neto et al., 2021).

In the beginning of the pandemic, there were reservations on the use of corticosteroids due concerns for prolonging viral shedding, which was seen in other viral diseases such as influenza. Since then, a European study on dexamethasone showed improved survival in patients requiring oxygen support (Recovery Collaborative Group et al., 2021). Methylprednisolone has been the preferred corticosteroid formulation in non-COVID-19 ARDS in the United States. In our initial study, it was associated with prolonged survival compared to no methylprednisolone (Go et al., 2021). Other immunomodulators, such as tocilizumab and baricitinib, have been used as inpatient treatment although they are not readily available and are expensive. ((Kalil et al., 2021; Recovery Collaborative Group, 2021).

In our institution, azithromycin 500 mg IV for 5 days and hydroxychloroquine 800 mg on day 1 followed by 400 mg once a day for day 2–5 were also given to hospitalized patients. Methylprednisolone, with dose and duration at the discretion of the provider, were given to patients who were sick or at a higher risk of dying.

Corticosteroids have become standard of care for COVID-19. We would like to determine if the addition of hydroxychloroquine and azithromycin to methylprednisolone is associated with improved survival compared to methylprednisolone alone.

## Methodology

### Eligibility criteria

Real world data was collected from Hackensack Meridian Health (HMH), a NJ health network comprising of thirteen hospitals on patients ≥18 years of age, and hospitalized for at least 2 days between 1 March 2020 and 15 June 2020 with severe COVID -19 Pneumonia.^1^ These patients had a positive SARS-CoV-2 PCR and had SpO2 <94% on room air at sea level, a respiratory rate >30 breaths/min, PaO2/FiO2 <300 mm Hg, or lung infiltrates >50%.

### Patient and public involvement

Patient and public were not involved in the design, conduction and dissemination of this study.

### Data collection

Demographic data such as age, gender, race, ethnicity, comorbidities, and sex were self-reported. Weight and height were measured. SARS-CoV-2 was detected in nasal swabs by RT-PCR. Routine blood tests included complete blood count (CBC), coagulation profile, complete metabolic profile (CMP), inflammatory markers [interleukin-6 (IL-6), C reactive protein (CRP), d-dimer, and ferritin], and arterial blood gas (ABG). Data was entered into Redcap and abstracted from June to December 2020.

### Outcomes

The primary outcomes are the associations of combinations of hydroxychloroquine, azithromycin, and methylprednisolone to in hospital survival. In the patients who received hydroxychloroquine, azithromycin, and methylprednisolone, we then wanted to compare the association of methylprednisolone dose to in hospital survival. The secondary outcome is a descriptive analysis of inflammatory markers in survivors versus non-survivors in those who received hydroxychloroquine, azithromycin, and methylprednisolone.

### Statistical analysis

This is a secondary analysis from a propensity score matched cohort study comparing in-hospital survival of COVID-19 admitted patients that were treated with methylprednisolone and no methylprednisolone. The patients were matched based on variables associated with mortality such as age (≥60 years vs. <60 years), body mass index (BMI) (≥30.0 kg/m^2^ vs. <30.0 kg/m^2^), sex (Male/Female), diabetes (Yes/No), hypertension (Yes/No), cancer (Yes/No), respiratory rate (RR)[ >22 breaths per minute (bpm) vs. ≤22 bpm], chronic kidney disease (Yes/No), low oxygen (oxygenation >94% vs. oxygenation≥94%), CRP (>20 mg/dl vs. ≤ 20 mg/dl), and quick sequential organ failure assessment (qSOFA) (score: 0,1,2,3). (Go et al., 2021).

A nearest-neighbor method was employed using a caliper of 0.20 to obtain the propensity matched sample. A post-match assessment of the distribution of propensity scores (or logit of propensity scores) and balance in the adjusted variables between the no methylprednisolone (NMP) and methylprednisolone using standardized difference and variance ratio were performed. This analysis and graphical displays were obtained by the ASSESS statement of PROC MATCH in SAS 9.4 (Go et al., 2021).

Categorical variables were presented as the frequency and percentage while continuous variables were presented as the median and interquartile range. Shapiro-Wilk test was used to assess normality of continuous variables. Comparison of matched continuous variables was performed using two-sided paired *t*-test or Wilcoxon signed-rank test, as appropriate. Comparison of paired categorical variables was performed using McNemar’s test or Bowker test of symmetry, as appropriate (Go et al., 2021).

Estimates of time to event (in hospital survival) were obtained using Kaplan-Meier method which reported median (95% CI), 60-days rates (95% CI), and the intervals were calculated using the arcsine square root transformation method (Go et al., 2021). To examine association of risk factor of interest, Cox proportional hazard (PH) regression analysis with robust covariance ^2^(sandwich estimator) to account paired observations was conducted and hazard ratios (HRs), 95% CIs, and *p* values were reported in all univariable and multivariable analysis from PROC PHREG. The PH assumption, critical in Cox regression, was evaluated using a Kolmogorov-type supremum test in ASSESS statement of PROC PHREG. If the PH assumption was violated, then a continuous variable which also violated the PH and its interaction with time were included in the model to adjust for the significant interaction with time to the risk of in-hospital mortality. In the multivariable analysis, all covariates (*p* < 0.10) were included in an initial full model fit and backward elimination selection procedure was performed until significant variables (at 5% levels) were retained (Go et al., 2021). To this final model, an interaction term of the non-PH (NPH) covariate and time to in hospital mortality and the NPH covariate were added to adjust for the NPH property of the methylprednisolone. In the multivariable analysis, all covariates (*p* < 0.10) were included in an initial full model fit and backward elimination selection procedure was performed until significant variables (at 5% levels) were retained (Go et al., 2021). To this final model, an interaction term of the non-PH (NPH) covariate and time to in hospital mortality and the NPH covariate were added to adjust for the NPH property of the methylprednisolone or azithromycin, hydroxychloroquine, and methylprednisolone. Optimal cutoff value of methylprednisolone dose was conducted using the Youden index method based on logistic regression of the binary outcome, in hospital survival, and total dose/absolute weight/day (Go et al., 2021).

## Results

Between March 4 and 15 June 2020, 2041 patients were flagged in the electronic health record with a diagnosis of COVID-19 and pneumonia. A total of 539 patients were excluded based on ineligibility criteria (<18 years of age, pregnant, other formulations of corticosteroids, or hospitalized for less than 2 days) A propensity score matched sample was constructed out of 759 patients [380 in no methylprednisolone and 379 in methylprednisolone]. After an examination of the proportional hazards assumption, methylprednisolone, and fractionated inspired oxygen (FiO2) significantly violated it (both with *p* < 0.0001). Data on P/F ratio was lacking; and FiO2 was used since 95% of patients had this data. The supremum test also indicated that non-proportionality was observed in other variables such as nursing home, lack of taste or smell, white blood cells<11,000 cells/ml, creatinine>1.5 ng/ml, respiratory rate >22 breaths per minute (BPM), hydroxychloroquine, methylprednisolone, calcium, and initial diastolic blood pressure. All variables with non-proportional hazard were adjusted using FiO2, as indicated above (Supplementary Table S1–S3) (Supplementary Figure S1,S2).

In both methylprednisolone and no methylprednisolone, the patients were admitted 5 days from the onset of symptoms. The methylprednisolone group had higher levels of CRP (12.64 mg/dl vs. 9.88 mg/dl *p* < 0.047) and required more patients higher oxygen support compared to no methylprednisolone (129 vs. 35 *p* < 0.0001). At that time, the use of non-invasive mechanical ventilation (BPAP or CPAP) was rare due to concerns of aerosolization of the virus.

**FIGURE 1:**
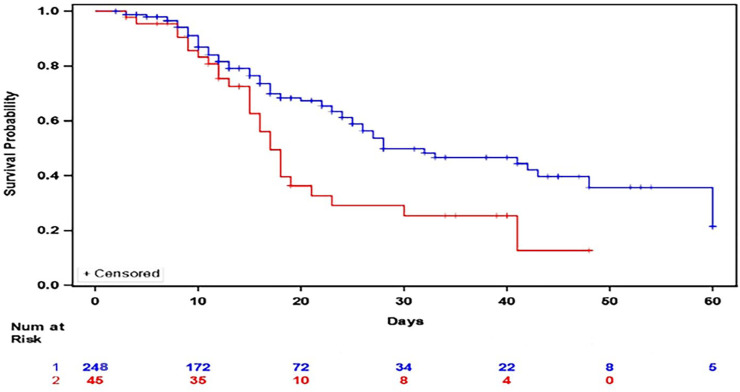
Kaplan Meier Plot of In Hospital Survival between patients who received methylprednisolone, hydroxychloroquine, and azithromycin (Blue) versus those who received methylprednisolone (Red).

Multivariate Cox regression shows that methylprednisolone, hydroxychloroquine, and azithromycin had prolonged survival compared to methylprednisolone alone [HR 0.45 (95% CI 0.22,0.91 *p* < 0.03)] (Figure 1). Nursing home residents [HR 2.77 (95% CI 1.67, 4.59 *p* < 0.0001)], coronary artery disease (CAD) [HR 2.93 (95% CI 1.31, 3.15 *p* = 0.001), and invasive mechanical ventilation [HR 3.02 (95% CI 1.71,5.34 *p* = 0.0001)] were associated with worse survival.

Other combinations were also explored. For those patients who received methylprednisolone and azithromycin, multivariate Cox regression showed improved survival compared to hydroxychloroquine (HR = 0.26; 95% CI 0.09,0.79 *p* = 0.015). For those who received methylprednisolone and hydroxychloroquine, multivariate Cox regression showed improved survival compared to azithromycin (HR 0.29; 95% CI 0.12,0.69 *p* = 0.0056). For those who received methylprednisolone, hydroxychloroquine, and azithromycin, they are associated with prolonged survival compared to methylprednisolone and hydroxychloroquine (HR = 0.52; 95% CI 0.32,0.83 *p* = 0.0072). Methylprednisolone, hydroxychloroquine, and azithromycin was associated with prolonged survival compared to hydroxychloroquine and azithromycin (HR 0.40 95% CI 0.24,0.66 *p* = 0.0004).

**FIGURE 2:**
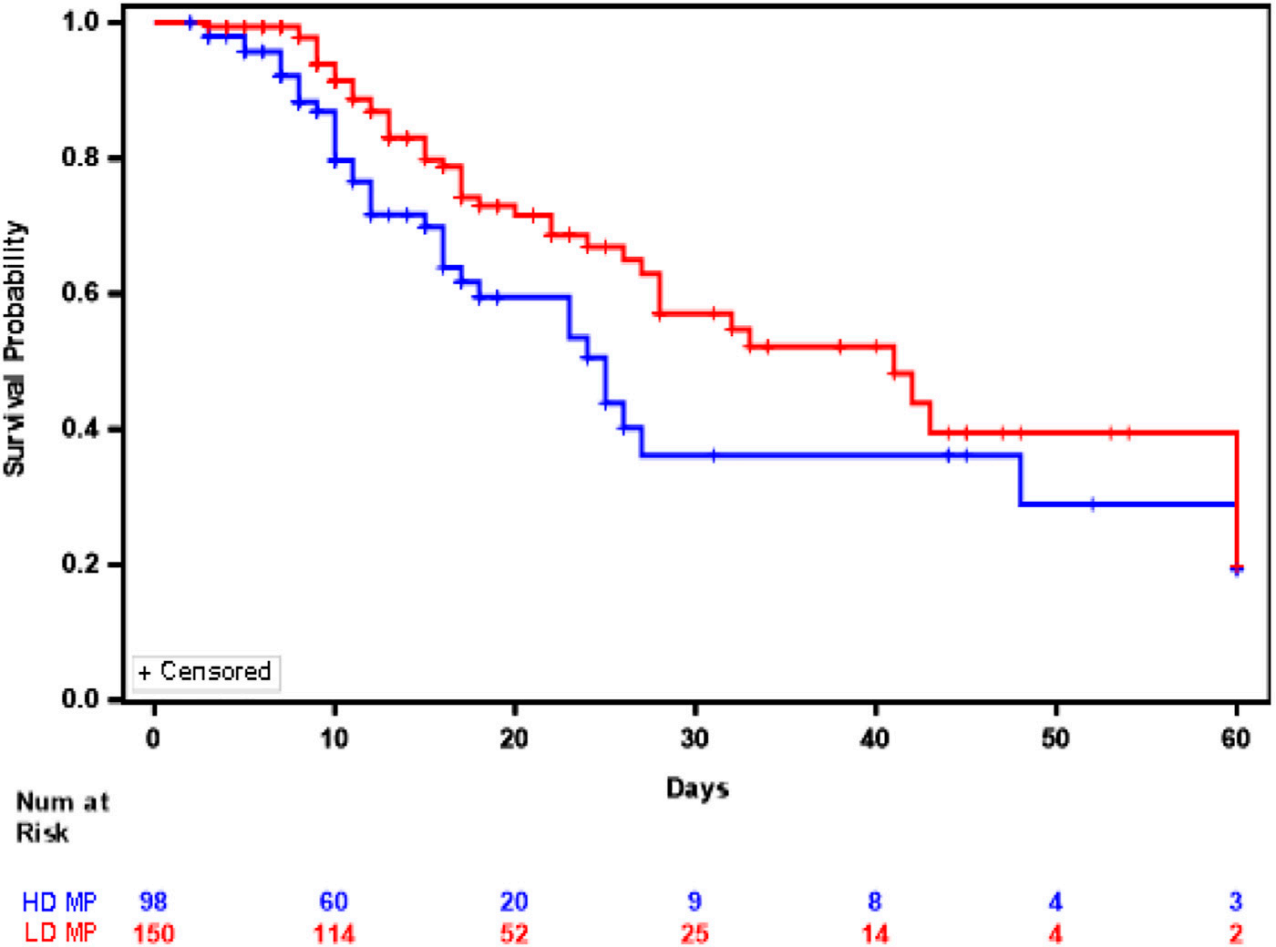
Kaplan Meier Plot of In Hospital Survival between patients who received high dose methylprednisolone, hydroxychloroquine, and azithromycin (Blue) versus those who received low dose methylprednisolone, hydroxychloroquine and azithromycin (Red).

We further wanted to see the effect of methylprednisolone dose combined with hydroxychloroquine and azithromycin. The Youden index method determined dose cutoff as 1.36 mg/kg/d (Go et al., 2021). Low-dose (LD) MP was defined as less than 1.36 mg/kg/d and high-dose (HD) MP was defined as greater than or equal to 1.36 mg/kg/d (Go et al., 2021). Out of the 248 COVID-19 patients that were treated with a combination of the three repurposed drugs methylprednisolone, hydroxychloroquine and azithromycin, 150 (60.5%) received low dose methylprednisolone and 95 (39.5%) received high dose methylprednisolone. Of the 150 patients that were treated with LD MP, 44 (29.3%) expired during their COVID-19 hospital stay. Of the 98 patients that were treated with HD MP, 36 (36.7%) expired during their COVID-19 hospital stay. The median in-hospital survival in the HD MP cohort was 25.0 days (95%CI:17.0–48.0 days) and median in-hospital survival in the LD MP cohort was 41.0 days (95% CI: 28 to N/A days), a difference in survival that were significantly different between the two cohorts (Log rank test *p* = 0.0250) (Figure 2). The Supremum test indicated that the assumption of proportional hazards was not violated (*p* = 0.3090), hence the Cox proportional hazard model was appropriate to analyze the comparison of High dose vs. Low Dose). Multivariate Cox regression showed that patients treated with high dose methylprednisolone, hydroxychloroquine, and azithromycin were associated 64% higher risk of in-hospital mortality compared to low dose methylprednisolone, hydroxychloroquine, and azithromycin (HR = 1.642; 95% CI 1.053 to 2.562; *p* = 0.0287).

In patients who received hydroxychloroquine, azithromycin, and methylprednisolone, the difference between survivors versus non-survivors for IL-6 (median 13.50 pg/ml versus 17 pg/mL *p* = 0.12), CRP (median 12.19 mg/ml versus 13.25 mg/mL *p* = 0.17), and ferritin (median 799.60 ug/L versus 867 ug/L *p* = 0.21) were not significant. The difference between survivors versus non-survivors for d-dimer (median 1.12 mcg/mL versus 1.91 mcg/mL *p* = 0.0007) was significant (Table 1). In this cohort, there were 47 thromboembolic diseases. The main cause of mortality was ARDS. There were no complications associated with hydroxychloroquine or azithromycin.

**TABLE 1:** Summary of Inflammatory markers by survival status of patients with severe. COVID-19 Pneumonia who received hydroxychloroquine, azithromycin, and methylprednisolone.

	Survivors	Non-survivors			
Variable	Median (IQR)	Min-Max	Median (IQR)	Min-Max	*p*-value
IL-6	13.50 (4,31)	(4,781)	17 (5.5,43.5)	(4.00,885.00)	0.1209
CRP	12.19 (6.82,18.60)	(0.41,38.00)	13.25 (7.8,20.8)	(1.00,38.00)	0.1677
D-dimer	1.12 (0.58,2.26)	(0.18,73.14)	1.91 (0.97,6.75)	(0.28,93.53)	**0.0007**
Ferritin	799.60 (326,1344)	(47,6451)	867 (375,1906)	(40,27,548)	0.2072

## Discussion

Combination therapy with hydroxychloroquine, azithromycin and methylprednisolone was associated with improved survival compared to methylprednisolone. There are two phases, a viral shedding phase, predominantly in the first week, followed by a hyperinflammatory phase, predominantly in the second week. These phases are not necessarily mutually exclusive. Infectious viral shedding may persist through the hyperinflammatory phase, with a median duration of 8 days and up to 15.2 days from onset of symptoms (van Kampen et al., 2021). This non-exclusivity may explain why there is added benefit to combination therapy rather than monotherapy of repurposed drugs, which typically target only one phase of the disease. Corticosteroids may still prolong viral shedding, which may be mitigated by hydroxychloroquine and azithromycin.

Therefore, timing of administration from onset of symptoms maybe important to see the efficacy of the repurposed drugs. Hydroxychloroquine with or without azithromycin maybe beneficial if given before 5–7 days from onset of symptoms. The randomized control trial of hydroxychloroquine with or without azithromycin in hospitalized patients with mild to moderate symptoms were recruited after at least 7 days from onset of symptoms (Cavalcanti et al., 2020). One of the strengths of this study is its low number of remdesivir, which has since become a standard of care (Recovery Collaborative Group et al., 2021; Gottlieb et al., 2022).

Interestingly, nursing home residents, CAD and invasive mechanical ventilation were independently associated with worse survival. Nursing home residents were associated with higher risk of death in other studies (Tang et al., 2020; Mehta et al., 2021). These maybe due to failure of recognition of disease, comorbidities, or ineffective triage early to hospitals (Mehta et al., 2021).

COVID-19 ARDS is associated with hypercoagulability. There is platelet and endothelial activation that may affect other organs and accelerate thrombotic complications in unknown and known CAD (Canzano et al., 2021). D-dimer, which is a marker of inflammation, thrombosis and hypercoagulability is most associated with persistence of symptoms and extra-pulmonary manifestations such as in multi-inflammatory syndrome in adults (MIS-A) (Patel et al., 2021). Unlike the other inflammatory markers in our study, only d-dimer was significantly different in non-survivors compared to survivors in those who received methylprednisolone, azithromycin, and hydroxychloroquine.

Interestingly, anti-platelet and anticoagulant therapy have not been associated with improved survival in those already requiring higher level of oxygen support (Chow et al., 2022; ATTACC Investigators, 2021; Goligher et al., 2021). This may be due to lung remodeling. The fibroproliferative phase of ARDS is associated with high levels of N-terminal peptide of type III procollagen (Burnham et al., 2013). This can occur as early as 72 h from the onset of ARDS (Burnham et al., 2013). There are currently no medications that directly reverse this remodeling.

Therefore, COVID-19 ARDS is not dissimilar to non-COVID-19 ARDS (Burnham et al., 2013; Brault et al., 2020; Haudebourg et al., 2020). With current strategies such as low tidal volume, neuromuscular blockers, and prone position, mortality was 34.9% (95% CI 31.4–38.5%) for mild ARDS, 40.3% (95% CI 37.4–43.4%) for moderate ARDS, and 46.1% (95% CI 41.9–50.4%) for severe ARDS (Milberg et al., 1995; Bellani et al., 2016). In our study, the common practice was to delay intubation due to shortage of mechanical ventilators and high risk of death once on invasive mechanical ventilators. Therefore, most patients already had severe ARDS once placed on mechanical ventilation. However, studies on timing to intubation did not affect mortality (Papoutsi et al., 2021; Al-Tarbsheh et al., 2022). This may be due to self-injurious lung injury in spontaneously breathing patients, that is akin to ventilator induced lung injury (Weaver et al., 2021).

### Limitations

Our study has several limitations. First, it is as an observational study. We tried to limit the known confounders via propensity score matching. Second, misclassification is possible due to manual extraction for medical health records although the data was reviewed multiple times by different reviewers. Third, methylprednisolone was given to patients who were at a higher risk of dying during the first pandemic surge. It is unknown if there is any association with survival in patients who were less sick or not on invasive mechanical ventilation. Fourth, there is a limited number of patients on non-invasive mechanical ventilation. While it may prevent or delay invasive mechanical ventilation, it is still controversial whether non-invasive mechanical ventilation improves mortality since it does not prevent self-inflicted lung injury (SILI). (Tobin et al., 2020; Grieco et al., 2021; Sullivan et al., 2022).

## Conclusion

Hydroxychloroquine, azithromycin, and methylprednisolone were associated with improved survival compared to methylprednisolone alone in hospitalized severe COVID-19 pneumonia. However, nursing home residents, CAD, and invasive mechanical ventilation were independently associated with mortality. Future implications of this study may warrant prospective studies evaluating the association of survival if hydroxychloroquine, azithromycin, and corticosteroids are given before the onset of ARDS, a symptom-based or phase-based approach to the administration of corticosteroids to hydroxychloroquine and azithromycin, or the use escalating d-dimer as biomarker for non-responsiveness to this combination.

## Data Availability

The original contributions presented in the study are included in the article/supplementary material, further inquiries can be directed to the corresponding author.

## Footnotes

1
www.covid19treatmentguidelines.nih.gov/management/clinical-management/hospitalized-adults–therapeutic-management/?utm_source=site&utm_medium=home&utm_campaign=highlights Accessed 11 March 2022.

2
www.who.int/publications/i/item/WHO-2019-nCoV-therapeutics-2022.2 Updated 3 March 2022. Accessed 11 March 2022.

## Abbreviations

ABG, arterial blood gas; ACE2, angiotensin converting enzyme 2; ARDS, acute respiratory distress syndrome; BMI, body mass index; BPM, breaths per minute; CAD, coronary artery disease; CBC, complete blood count; COVID-19, coronavirus disease 2019; CMP, complete metabolic profile; CRP, c reactive protein; FiO2, fractionated inspiratory oxygen; HDMP, high dose methylprednisolone; HMH, Hackensack Meridian Health; HR, hazard ratio; IL-6, interleukin—6; IQR, interquartile range; LDMP, low dose methylprednisolone; MIS-A, Multi-inflammatory syndrome in Adults; NMP, No methylprednisolone; NPH, Non-proportional hazardP/F PaO2/FiO2; PH, Proportional Hazard; qSOFA, quick sequential organ failure assessment; RR, respiratory rate; SILI, Self-inflicted lung injury.

## References

[B1] Al-Tarbsheh A. Chong W. Oweis J. Saha B. Feustel P. Leamon A. (2022). Clinical outcomes of early versus late intubation in COVID-19 patients. Cureus 14 (1), e21669. 10.7759/cureus.21669 35237472 PMC8882044

[B2] ATTACC Investigators (2021). ATTACC Investigators et al.Therapeutic Anticoagulation with Heparin in Critically Ill Patients with Covid-19. N. Engl. J. Med. 385 (9), 777. 34351722 10.1056/NEJMoa2103417 PMC8362592

[B3] Badu K. Oyebola K. Zahouli J. Z. B. Fagbamigbe A. F. de Souza D. K. Dukhi N. (2021). SARS-CoV-2 viral shedding and transmission dynamics: Implications of WHO COVID-19 discharge guidelines. Front. Med. 8, 648660. 10.3389/fmed.2021.648660 PMC8259580 34239886

[B4] Bellani G. Laffey J. G. Pham T. Fan E. Brochard L. Esteban A. (2016). Epidemiology, patterns of care, and mortality for patients with acute respiratory distress syndrome in intensive care units in 50 countries. JAMA 315 (8), 788–800. 10.1001/jama.2016.0291 26903337

[B5] Brault C. Zerbib Y. Kontar L. Fouquet U. Carpentier M. Metzelard M. (2020). COVID-19- versus non-COVID-19-related acute respiratory distress syndrome: Differences and similarities. Am. J. Respir. Crit. Care Med. 202 (9), 1301–1304. 10.1164/rccm.202005-2025LE 32857595 PMC7605202

[B6] Burnham E. L. Janssen W. J. Riches D. W. H. Moss M. Downey G. (2013). The fibroproliferative response in acute respiratory distress syndrome: Mechanisms and clinical significance. Eur. Respir. J. 32, 276–285. 10.1183/09031936.00196412 PMC4015132 23520315

[B7] Canzano P. Brambilla M. Porro B. Cosentino N. Tortorici E. Vicini S. (2021). Platelet and endothelial activation as potential mechanisms behind the thrombotic complications of COVID-19 patients. JACC. Basic Transl. Sci. 6 (3), 202–218. 10.1016/j.jacbts.2020.12.009 33649738 PMC7904280

[B8] Cavalcanti A. B. Zampieri F. G. Rosa R. G. Azevedo L. C. P. Veiga V. C. Avezum A. (2020). Hydroxychloroquine with or without azithromycin in mild-to-moderate covid-19. N. Engl. J. Med. 383 (21), 2041–2052. 10.1056/NEJMoa2019014 32706953 PMC7397242

[B9] Chen F. Chan K. H. Jiang Y. Kao R. Y. T. Lu H. T. Fan K. W. (2004). *In vitro* susceptibility of 10 clinical isolates of SARS coronavirus to selected antiviral compounds. J. Clin. Virol. 31 (1), 69–75. 10.1016/j.jcv.2004.03.003 15288617 PMC7128415

[B10] Chow J. H. Rahnavard A. Gomberg-Maitland M. Chatterjee R. Patodi P. Yamane D. P. (2022). Association of early aspirin use with in-hospital mortality in patients with moderate COVID-19. JAMA Netw. Open 5 (3), e223890. 10.1001/jamanetworkopen.2022.3890 35323950 PMC8948531

[B11] Echeverría-Esnal D. Martin-Ontiyuelo C. Navarrete-Rouco M. E. De-Antonio Cuscó M. Ferrández O. Horcajada J. P. (2021). Azithromycin in the treatment of COVID-19: A review. Expert Rev. anti. Infect. Ther. 19 (2), 147–163. 10.1080/14787210.2020.1813024 32853038

[B12] Fiolet T. Guihur A. Rebeaud M. E. Mulot M. Peiffer-Smadja N. Mahamat-Saleh Y. (2021). Effect of hydroxychloroquine with or without azithromycin on the mortality of coronavirus disease 2019 (COVID-19) patients: A systematic review and meta-analysis. Clin. Microbiol. Infect. 27 (1), 19–27. 10.1016/j.cmi.2020.08.022 32860962 PMC7449662

[B13] Go R. C. Shah R. Nyirenda T. (2021). Methylprednisolone and 60 Days in hospital survival in coronavirus disease 2019 pneumonia. Crit. Care Explor, 3. e0493. 10.1097/CCE.0000000000000493 34291223 PMC8291358

[B14] Go R. C. (2021). “Chapter 4: Repurposing therapies for COVID-19,” in Understanding crisis in critical care. Editor GoMc Graw Hill R. C. NYC.

[B15] Goligher E. C. Bradbury C. A. McVerry B. J. Lawler P. R. Berger J. S. Gong M. N. (2021). Therapeutic anticoagulation with heparin in noncritically ill patients with covid-19. N. Engl. J. Med. Overseas. Ed. 385 (9), 790–802. 10.1056/nejmoa2105911 PMC8362594 34351721

[B16] Gottlieb R. L. Vaca C. E. Paredes R. Mera J. Webb B. J. Perez G. (2022). Early remdesivir to prevent progression to severe COVID-19 in outpatients. N. Engl. J. Med. 386, 305–315. 10.1056/NEJMoa2116846 34937145 PMC8757570

[B17] Grieco D. L. Maggiore S. M. Roca O. Spinelli E. Patel B. K. Thille A. W. (2021). Non-invasive ventilatory support and high-flow nasal oxygen as first-line treatment of acute hypoxemic respiratory failure and ARDS. Intensive Care Med. 47, 851–866. 10.1007/s00134-021-06459-2 34232336 PMC8261815

[B18] Haudebourg A. F. Perier F. Tuffet S. de Prost N. Razazi K. Mekontso Dessap A. (2020). Respiratory mechanics of COVID-19- versus non-COVID-19-associated acute respiratory distress syndrome. Am. J. Respir. Crit. Care Med. 202 (2), 287–290. 10.1164/rccm.202004-1226LE 32479162 PMC7365370

[B19] Kalil A. C. Patterson T. F. Mehta A. K. Tomashek K. M. Wolfe C. R. Ghazaryan V. (2021). Baricitinib plus remdesivir for hospitalized adults with covid-19. N. Engl. J. Med. 384 (9), 795–807. 10.1056/NEJMoa2031994 33306283 PMC7745180

[B20] Liu J. Cao R. Xu M. Wang X. Zhang H. Hu H. (2020). Hydroxychloroquine, a less toxic derivative of chloroquine, is effective in inhibiting SARS-CoV-2 infection *in vitro*. Cell Discov. 6, 16. 10.1038/s41421-020-0156-0 32194981 PMC7078228

[B21] Mehta H. B. Li S. Goodwin J. S. (2021). Risk factors associated with SARS-CoV-2 infections, hospitalization, and mortality among US nursing home residents. JAMA Netw. Open 4 (3), e216315. 10.1001/jamanetworkopen.2021.6315 33787905 PMC8013796

[B22] Milberg J. A. Davis D. R. Steinberg K. P. Hudson L. D. (1995). Improved survival of patients with acute respiratory distress syndrome (ARDS): 1983-1993. JAMA 273 (4), 306–309. 10.1001/jama.1995.03520280052039 7815658

[B23] Papoutsi E. Giannakoulis V. G. Xourgia E. Routsi C. Kotanidou A. Siempos I. I. (2021). Effect of timing of intubation on clinical outcomes of critically ill patients with COVID-19: A systematic review and meta-analysis of non-randomized cohort studies. Crit. Care 25, 121. 10.1186/s13054-021-03540-6 33766109 PMC7993905

[B24] Patel P. DeCuir J. Abrams J. Campbell A. P. Godfred-Cato S. Belay E. D. (2021). Clinical characteristics of multisystem inflammatory syndrome in adults: A systematic review. JAMA Netw. Open 4 (9), e2126456. 10.1001/jamanetworkopen.2021.26456 34550381 PMC8459192

[B25] Réa-Neto Á. Bernardelli R. S. Câmara B. M. D. Reese F. B. Queiroga M. V. O. Oliveira M. C. (2021). An open-label randomized controlled trial evaluating the efficacy of chloroquine/hydroxychloroquine in severe COVID-19 patients. Sci. Rep. 11, 9023. 10.1038/s41598-021-88509-9 33907251 PMC8079411

[B26] Recovery Collaborative Group. Horby P. Mafham M. Linsell L. Bell J. L. Staplin N. (2020). Effect of hydroxychloroquine in hospitalized patients with covid-19. N. Engl. J. Med. 383 (21), 2030–2040. 10.1056/NEJMoa2022926 33031652 PMC7556338

[B27] Recovery Collaborative Group. Horby P. Lim W. S. Emberson J. R. Mafham M. Bell J. L. (2021). Dexamethasone in hospitalized patients with covid-19. N. Engl. J. Med. 384 (8), 693–703. 10.1056/NEJMoa2021436 32678530 PMC7383595

[B28] Recovery Collaborative Group (2021). Tocilizumab in patients admitted to hospital with COVID-19 (RECOVERY): A randomised, controlled, open-label, platform trial. Lancet 397 (10285), 1637–1645. 10.1016/S0140-6736(21)00676-0 33933206 PMC8084355

[B29] Rosenberg E. S. Dufort E. M. Udo T. Wilberschied L. A. Kumar J. Tesoriero J. (2020). Association of treatment with hydroxychloroquine or azithromycin with in-hospital mortality in patients with COVID-19 in New York state. JAMA. 323 (24), 2493–2502. 10.1001/jama.2020.8630 32392282 PMC7215635

[B30] Sullivan Z. P. Zazzeron L. Berra L. (2022). Noninvasive respiratory support for COVID-19 patients: When, for whom, and how?., J. intensive care. 10 3. 10.1186/s40560-021-00593-1 35033204 PMC8760575

[B31] Tang O. Bigelow B. F. Sheikh F. Peters M. Zenilman J. M. Bennett R. (2020). Outcomes of nursing home COVID-19 patients by initial symptoms and comorbidity: Results of universal testing of 1970 residents. J. Am. Med. Dir. Assoc. 21 (12), 1767–1773. 10.1016/j.jamda.2020.10.011 33153910 PMC7556822

[B32] Tobin M. J. Laghi F. Jubran A. (2020). P-SILI is not justification for intubation of COVID-19 patients. *Ann. Intensive Care* . Ann. Intensive Care 105, 24. 10.1186/s13613-020-00724-1 PMC7397710 32748116

[B33] van Kampen J. J. A. van de Vijver D. A. M. C. Fraaij P. L. A. Haagmans B. L. Lamers M. M. Okba N. (2021). Duration and key determinants of infectious virus shedding in hospitalized patients with coronavirus disease-2019 (COVID-19). Nat. Commun. 12, 267. 10.1038/s41467-020-20568-4 33431879 PMC7801729

[B34] Vincent M. J. Bergeron E. Benjannet S. (2005). Chloroquine is a potent inhibitor of SARS coronavirus infection and spread. Virol. J. 2, 69. 10.1186/1743-422X-2-69 16115318 PMC1232869

[B35] Wang M. Cao R. Zhang L. (2020). Remdesivir and chloroquine effectively inhibit the recently emerged novel coronavirus (2019-nCoV) *in vitro* . Cell Res. 30, 269–271. 10.1038/s41422-020-0282-0 32020029 PMC7054408

[B36] Weaver L. Das A. Saffaran S. Yehya N. Scott T. E. Chikhani M. (2021). High risk of patient self-inflicted lung injury in COVID-19 with frequently encountered spontaneous breathing patterns: A computational modelling study. Ann. Intensive Care 11, 109. 10.1186/s13613-021-00904-7 34255207 PMC8276227

[B37] World Health Organization (2021a). World health organization (WHO) coronavirus disease 19 dashboard. Available at: https://covid19.who.int/ (Accessed June 2, 2021).

[B38] World Health Organization (2021b). World health organization (WHO) coronavirus disease 19 dashboard. Available at: https://covid19.who.int/ (Accessed March 13, 2021).

[B39] Yao X. Ye F. Zhang M. Cui C. Huang B. Niu P. (2020). *In vitro* antiviral activity and projection of optimized dosing design of hydroxychloroquine for the treatment of severe acute respiratory syndrome coronavirus 2 (SARS-CoV-2). Clin. Infect. Dis. 71 (15), 732–739. 10.1093/cid/ciaa237 32150618 PMC7108130

[B40] Zhou P. Yang X-L. Wang X-G. (2020). A pneumonia outbreak associated with a new coronavirus of probable bat origin. Nature 579, 270. 10.1038/s41586-020-2012-7 32015507 PMC7095418

