# Supplementary material for: Hydroxychloroquine, azithromycin and methylprednisolone and in hospital survival in severe COVID-19 pneumonia
Source: Front Pharmacol. 2022 Sep 27;13:935370. doi: 10.3389/fphar.2022.935370 (PMC9551186; doi:10.3389/fphar.2022.935370)
Supplement: Supplementary file 1 [file DataSheet1.PDF]

Supplemental Section for **Hydroxychloroquine, Azithromycin and Methylprednisolone and In Hospital Survival in Severe COVID-19 Pneumonia**

Ronaldo C. Go and Themba Nyirenda

Table of Contents

Table S1: Baseline Demographics Disease Characteristics of Unmatched Population and Propensity Matched Population……………………………………………….……………..3
Table S2: Standardized Mean Differences (Methylprednisolone – No Methylprednisolone)..….5
Figure S1: Plot of Differences Methylprednisolone – No Methylprednisolone in Hospitalized COVID-19 Patients……...………..……………...………..………………………………6
Figure S2: LPS Cloud Plots showing Distributions of Logit of Propensity Scores for MP and NMP treated COVID-19 patients……………………………...……………………………..7
Table S3: Frequencies of Hydroxychloroquine, Azithromycin, and Methylprednisolone………8

Table S1. Baseline Demographics Disease Characteristics on Unmatched Population andPropensity Score Matched Population. (17)

Variable	Unmatched No methylprednisolone (N=645)	Unmatched Methylprednisolone (N=476)	P Value	Propensity score matched No methylprednisolone (N = 380)	Propensity Score matched Methylprednisolone (N = 379)	P Value
Age in years	64.00(53.00,79.00)	64.00(56.00,73.00)	0.6620	65.00(54.00,80.00)	64.00(55.00,74.00)	0.1129
Male	411(63.43)	219(46)	0.6079	238(62.6)	244 (64.21)	0.5175
Weight (kg)	81.20(68.70,90.72)	83.90(71.10,99.80)	0.0040	81.67(70.30,95.85)	83.90(71.45,99.80)	0.4270
BMI (kg/m^2)	28.18(24.83,31.65)	29.48(25.94,34.30)	<.0001	29.82(25.51,32.80)	29.69(25.82,34.31)	0.1912
White	336(51.85)	154(32.35)	0.6079	198(52)	193(51)	0.5085
Asian	45(6.94)	31(6.51)	0.6079	20(5.26)	28(7.4)	0.5085
Black	91(14.04)	47(9.87)	0.6079	57(15)	42(11.1)	0.5085
Other	154(23.77)	229(48.11)	0.6079	94(24.7)	96(25)	0.5085
Community	527(81.33)	250(52.52)	<.0001	309(81.3)	220(58)	**<.0001**
Academic	121(18.67)	174(36.55)	<.0001	74(19.4)	160(42)	**<.0001**
Nursing Home	125(19.38)	43(9.03)	<.0001	308(81.1)	340(89.7)	**0.0002**
Former/Current smoker	116(17.98)	96(20.17)	0.0377	77(20.3)	88(23)	0.2334
Never smoker	468(72.56)	276(57.98)	0.0377	280(73.7)	253(66)	0.2334
SOB	412(63.88)	334(70.17)	<.0001	249(65.5)	298(78.63)	**<.0001**
Cough	417(64.65)	301(63.24)	0.0327	244(64.2)	271(71.5)	**0.0303**
AMS	109(16.99)	46(9.66)	0.0058	63(16.6)	41(10.8)	**0.0032**
GI	149(23.1)	88(18.49)	0.4077	76(20)	81(21.37)	0.6545
Anosmia or Ageusia	10(1.55)	9(1.89)	0.4834	6(1.58)	9(2.4)	0.4445
Duration of Symptoms prior to admission	5.00(2.00,7.00)	5.00(3.00,7.00)	0.0165	5.00(2.00,7.00)	5.00(3.00,7.00)	0.0523
Diabetes	214(33.18)	157(32.98)	0.1888	144(37.9)	139(36.6)	0.8221
COPD	27(4.19)	30(6.3)	0.0506	20(5.26)	28(7.37)	0.2360
Asthma	40(6.22)	40(8.4)	0.0569	24(6.27)	37(9.76)	0.0832
Cancer	61(9.46)	49(10.29)	0.2594	43(11.3)	43(11.32)	1.0000
CAD	86(13.33)	67(14.08)	0.2839	50(13.15)	61(16)	0.2594
CVA	28(4.34)	15(3.15)	0.6336	18(4.71)	14(3.69)	0.5887
CHF	51(7.91)	32(6.72)	0.9071	18(4.71)	28(7.37)	1.0000
Arrhythmia	65(10.1)	38(7.98)	0.5967	41(10.79)	30(7.91)	0.2126
Chronic Kidney Disease	40(6.23)	33(6.93)	0.3249	28(7.36)	31(8.16)	0.6862
Rheumatologic Disease	15(2.33)	22(4.62)	0.0159	10(2.63)	19(5.01)	0.0909
qSOFA 0	375(58.14)	225(47.27)	0.2544	224(58.9)	222(58.5)	0.7647
qSOFA 1	199(30.85)	156(32.77)	0.2544	130(34.2)	130(34.3)	0.7647
qSOFA 2	51(7.9)	31(6.51)	0.2544	28(7.36)	26(6.86)	0.7647
qSOFA 3	4(0.64)	2(0.42)	0.2544	1(0.26)	2(0.53)	0.7647
O2 sat < 94%	305(47.3)	225(47.27)	0.0069	216(56.84)	218(57.5)	0.8265
Temperature	99.00(98.00,100.70)	99.30(98.00,100.80)	0.0476	98.80(97.70,100.40)	99.25(98.00,100.65)	**0.0027**
Heart Rate	95.00(82.00,108.00)	95.00(82.00,108.00)	0.0581	95.00(84.00,108.00)	97.00(86.00,108.00)	0.2467
Respiratory Rate	19.00(18.00,21.00)	20.00(18.00,22.00)	0.0097	19.00(18.00,22.00)	20.00(18.00,22.00)	0.2622
Nasal Cannula	227(35.19)	137(28.78)	0.0005	161(42.36)	132(35)	**0.0045**
Venti mask	4(.62)	3(0.63)	0.0005	2(.5)	3(0.8)	**0.0045**
High Flow	8(1.2)	15(3.15)	0.0005	6(1.6)	15(4)	**0.0045**
CPAP	1(0.15)	2(0.42)	0.0005	1(.26)	2(.5)	**0.0045**
BPAP	0(0.00)	2(0.42)	0.0005	0(0.00)	2(.5)	**0.0045**
Invasive Mechanical Ventilation	55(8.5)	138(28.99)	<.0001	35(9.2)	129(34)	**<.0001**
WBC	6.50(5.00,9.10)	6.50(5.10,9.50)	0.5947	6.60(5.10,9.20)	6.50(5.10,9.55)	0.8365
HGB	13.40(12.00,14.50)	13.50(12.20,14.80)	0.2190	13.40(12.20,14.50)	13.50(12.20,14.70)	0.5022

PLT	200.00(158.00,251.00)	186.00(147.00,251.00)	0.0596	203.00(161.00,259.00)	189.50(147.00,252.00	**0.0238**
ALC	0.90(0.60,1.20)	0.79(0.60,1.10)	0.0007	0.90(0.60,1.30)	0.80(0.60,1.10)	**0.0004**
IL6	11.50(5.00,34.00)	12.00(5.00,32.00)	0.6607	12.50(5.00,37.50)	12.00(5.00,31.50)	0.8044
CRP	10.91(5.20,20.79)	13.11(7.09,20.20)	0.0444	11.71(5.34,22.10)	13.40(7.10,20.34)	0.3187
D-Dimer	1.01(0.64,2.11)	0.98(0.61,1.89)	0.7909	1.01(0.65,2.07)	0.98(0.61,1.91)	0.8118
Ferritin	641.89(320.65,1453.60	838.96(430.40,1569.80)	0.0044	727.45(331.61,1470.50)	853.21(444.90,1569.80)	**0.0231**
Creatinine	1.00(0.80,1.40)	1.01(0.80,1.33)	0.9379	1.01(0.80,1.49)	1.01(0.80,1.35)	0.2327
Troponin	0.03(0.01,0.30)	0.02(0.01,0.09)	0.0516	0.03(0.01,0.30)	0.02(0.01,0.09)	0.1355
BNP	103.70(29.85,701.30)	88.80(26.20,362.00)	0.1702	129.70(40.60,941.10)	85.25(25.60,339.35)	**0.0141**
Hydroxychloroquine	463(73.73)	333(69.96)	<.0001	270(71.1)	318(84)	**<.0001**
Azithromycin	438(71.78)	277(58.19)	0.2793	256(67.4)	264(70)	0.1672
Remdesivir	4(0.62)	63(13.24)	0.0061	3(0.78)	10(2.6)	0.0512
Tocilizumab	31(4.8)	11(2.31)	<.0001	14(3.68)	64(16.8)	**<.0001**
Convalescent Plasma	0(0.00)	4(0.84)	0.0002	0(0.00)	4(1)	**0.0015**
ECMO	1(0.17)	9(1.9)	0.0011	1(0.26)	9(2.4)	**0.0104**
Dialysis	19(2.95)	11(2.3)	0.8748	14(3.78)	11(2.9)	0.6853

HR = Hazard Ratio; CI = Confidence Interval; SOB = Shortness of Breath; AMS = Altered Mental Status; GI = Gastrointestinal Symptoms; PTA = Prior to admission; COPD = Chronic Obstructive Disease; CAD = Coronary Artery Disease; CVA = Cerebrovascular Accident; CHF = Congestive Heart Failure; LFTs = elevated liver function tests; qSOFA = Quick Sepsis Related Organ Failure Assessment; HCQ = Hydroxychloroquine; AZ = Azithromycin; MP = Methylprednisolone; HD MP = High Dose Methylprednisolone; LD MP = Low Dose Methylprednisolone; WBC= White Blood Cells; HGB = Hemoglobin; PLT = Platelet; ALC = Absolute Lymphocyte Count; ECMO =Extracorporeal Membrane Oxygenation

Table S2. Standardized Mean Differences (Methylprednisolone – No Methylprednisolone. (17)

Variable	Observations	Mean Difference	Standard Deviation	Standardized Difference	Percent Reduction	Variance Ratio
Logit Prop Score	All	0.18717	0.432253	0.43300		1.1296
	Region	0.18546		0.42905	0.91	1.1395
	Matched	0.01401		0.03242	92.51	1.0738
Obesity Status (BMI ≥ 30.0 kg/m^2)	All	-0.12778	0.490334	-0.26060		1.0820
	Region	-0.12719		-0.25940	0.46	1.0812
	Matched	-0.00781		-0.01593	93.89	1.0016
Older Age (>60 years)	All	-0.03502	0.483048	-0.07249		0.9623
	Region	-0.03565		-0.07381	0.00	0.9618
	Matched	0.02604		0.05391	25.63	1.0353
Sex	All	-0.00812	0.480332	-0.01690		0.9903
	Region	-0.00871		-0.01813	0.00	0.9896
	Matched	-0.02083		-0.04337	0.00	0.9765
Diabetes	All	-0.04801	0.477468	-0.10054		1.0636
	Region	-0.04747		-0.09942	1.12	1.0628
	Matched	0.00781		0.01636	83.73	0.9914
Hypertension	All	-0.05334	0.495965	-0.10755		0.9754
	Region	-0.05248		-0.10581	1.62	0.9756
	Matched	0.01042		0.02100	80.47	1.0069
Cancer	All	-0.02157	0.308368	-0.06994		1.1960
	Region	-0.02141		-0.06944	0.72	1.1942
	Matched	0.00000		0.00000	100.00	1.0000
Resp. Rate>22 bpm	All	-0.06401	0.396891	-0.16127		1.2802
	Region	-0.06374		-0.16059	0.42	1.2785
	Matched	-0.00781		-0.01968	87.79	1.0263
Renal failure	All	-0.01252	0.259765	-0.04821		1.1722
	Region	-0.01241		-0.04779	0.86	1.1704
	Matched	-0.00781		-0.03008	37.62	1.0978
Oxygen <94%	All	-0.10506	0.496524	-0.21158		0.9776
	Region	-0.10428		-0.21003	0.73	0.9775
	Matched	-0.01042		-0.02098	90.08	0.9943
CRP >20 ng/L	All	-0.06192	0.389826	-0.15884		1.2908
	Region	-0.06166		-0.15818	0.41	1.2891
	Matched	-0.01823		-0.04676	70.56	1.0699
Standard deviation of All observations used to compute standardized differences						

Figure S1. Plot of differences Methylprednisolone – No Methylprednisolone in hospitalizedCOVID-19 patients. The plot shows that the differences were very close to zero as were the Logit propensity scores. (17)

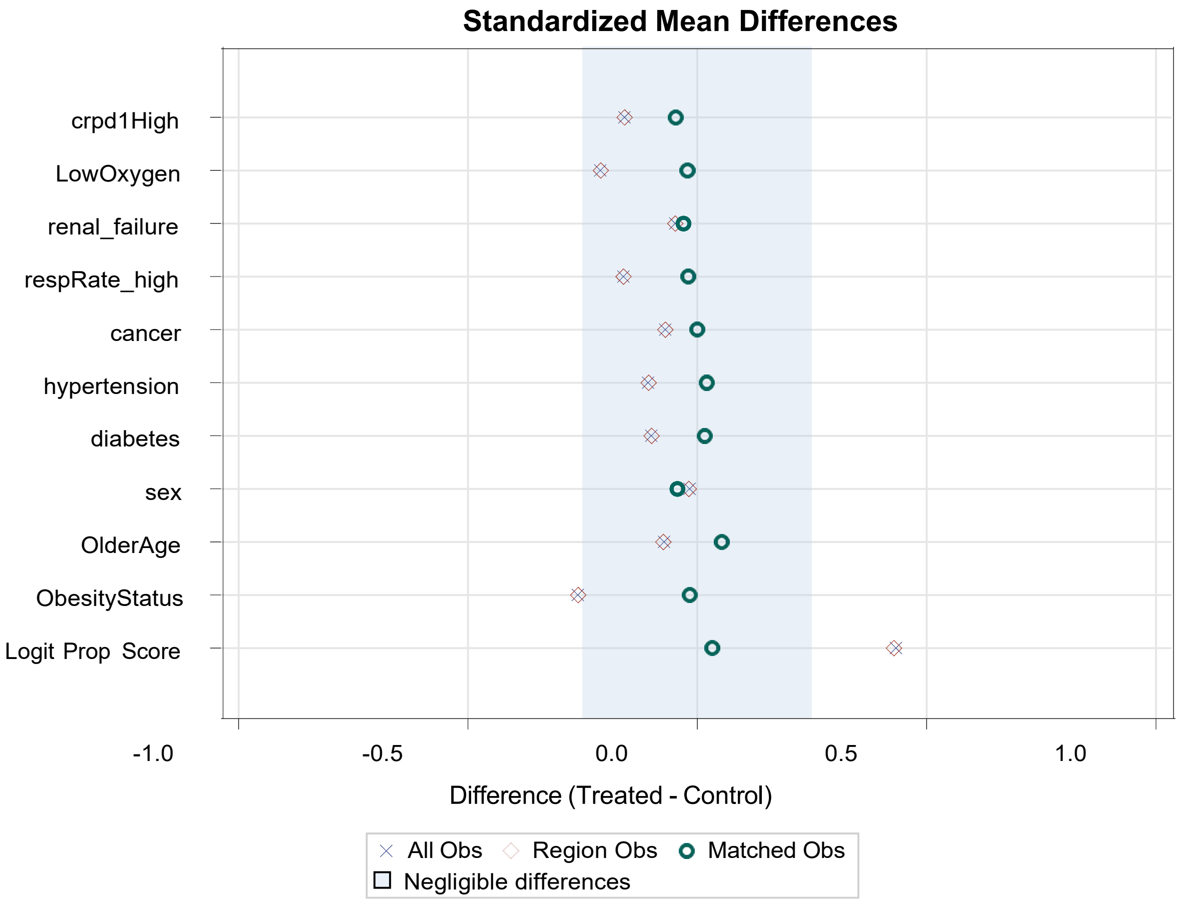

Figure S2 LPS Cloud Plots showing Distributions of Logit of Propensity Scores for MP and NMP treated COVID-19 patients. The propensity matching procedure failed to match only 8 ofthe methylprednisolone patients with any of patients who did not receive methylprednisolone during their COVID-19 hospitalization. (17)

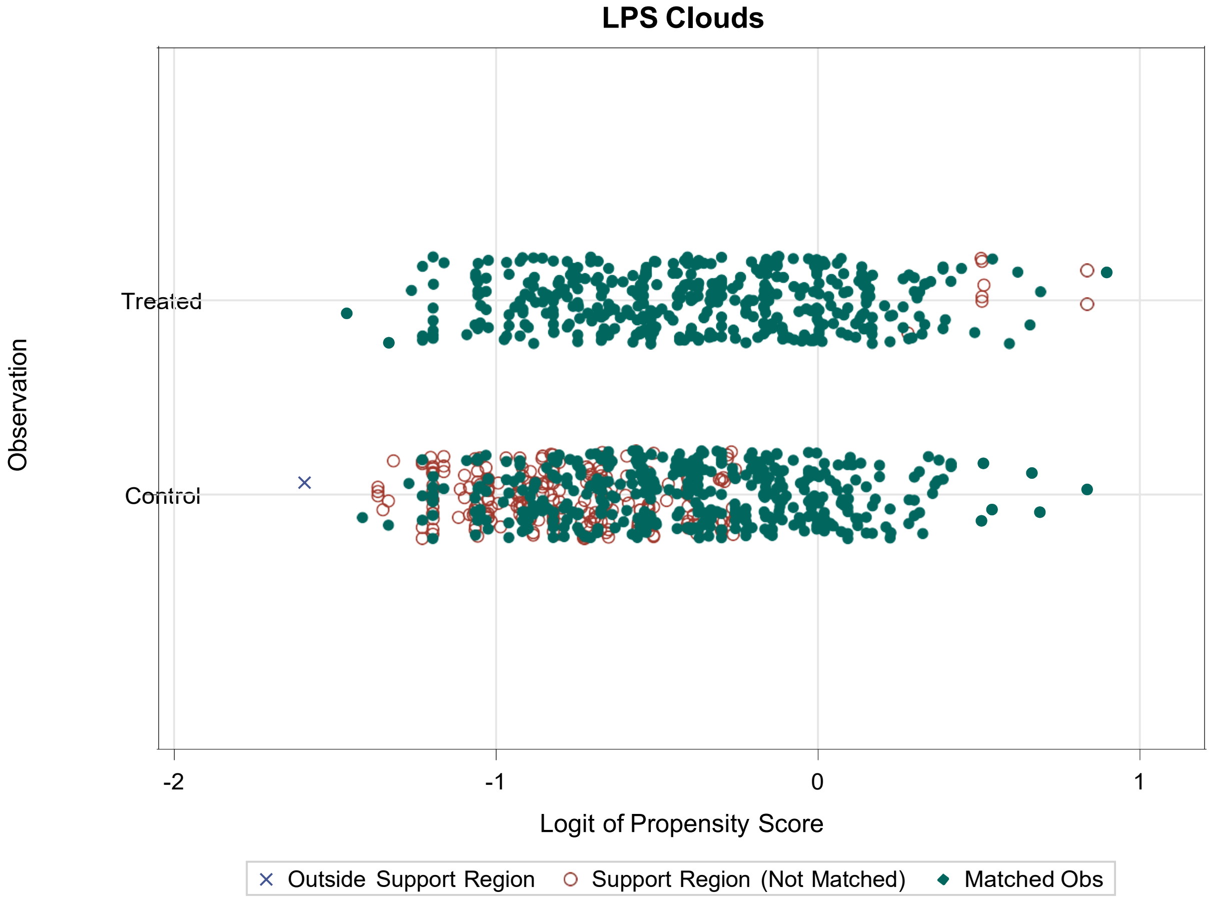

Table S3. Frequencies of Hydroxychloroquine, Azithromycin, and Methylprednisolone

Drug Combination	Frequency	Percent	Cumulative Frequency	Cumulative Percent
**No methylprednisolone, No hydroxychloroquine, No azithromycin**	58	7.64	58	7.64
**hydroxychloroquine**	67	8.83	125	16.47
**azithromycin**	54	7.11	179	23.58
**hydroxychloroquine And azithromycin**	201	26.48	380	50.07
**methylprednisolone**	45	5.93	425	55.99
**methylprednisolone, hydroxychloroquine**	70	9.22	495	65.22
**methylprednisolone, azithromycin**	16	2.11	511	67.33
**methylprednisolone, hydroxychloroquine, and azithromycin**	248	32.67	759	100.00
